# Beyond BMI for self-estimates of body size and shape: A new method for developing stimuli correctly calibrated for body composition

**DOI:** 10.3758/s13428-020-01494-1

**Published:** 2020-10-13

**Authors:** Nadia Maalin, Sophie Mohamed, Robin S. S. Kramer, Piers L. Cornelissen, Daniel Martin, Martin J. Tovée

**Affiliations:** 1 School of Psychology, University of Lincoln, Lincoln, UK; 2 Department of Psychology, Northumbria University, Newcastle upon Tyne, UK; 3 School of Sport and Exercise Science, University of Lincoln, Lincoln, UK

**Keywords:** Body image, Body shape, Body mass index, Body composition, Muscle content, Adiposity

## Abstract

Accurate self-assessment of body shape and size plays a key role in the prevention, diagnosis, and treatment of both obesity and eating disorders. These chronic conditions cause significant health problems, reduced quality of life, and represent a major problem for health services. Variation in body shape depends on two aspects of composition: adiposity and muscularity. However, most self-assessment tools are unidimensional. They depict variation in adiposity only, typically quantified by the body mass index. This can lead to substantial, and clinically meaningful, errors in estimates of body shape and size. To solve this problem, we detail a method of creating biometrically valid body stimuli. We obtained high-resolution 3D body shape scans and composition measures from 397 volunteers (aged 18–45 years) and produced a statistical mapping between the two. This allowed us to create 3D computer-generated models of bodies, correctly calibrated for body composition (i.e., muscularity and adiposity). We show how these stimuli, whose shape changes are based on change in composition in two dimensions, can be used to match the body size and shape participants believe themselves to have, to the stimulus they see. We also show how multivariate multiple regression can be used to model shape change predicted by these 2D outcomes, so that participants’ choices can be explained by their measured body composition together with other psychometric variables. Together, this approach should substantially improve the accuracy and precision with which self-assessments of body size and shape can be made in obese individuals and those suffering from eating disorders.

## Introduction

The accurate perception and indexing of body adiposity, whether it is too low or too high, is a vital health prevention and management goal. There has been an inexorable worldwide rise in obesity with a concomitant pressure on public health resources (Ogden et al. 2006; Swinburn et al., 2011). At least 2.1 billion people (30% of the global population) are thought to be overweight or obese, and 5% of deaths worldwide can be directly attributed to obesity (Dobbs et al., 2014; Tremmel, Gerdtham, Nillson, & Saha, 2017). Obesity can take up to 8 years off a person’s life expectancy and causes decades of ill health (Grover et al., 2014). Additionally, obesity costs the world economy at least $2.0 trillion or 2.8% of the global domestic product (Dobbs et al., 2014). A potential contributory factor to the rise in obesity is the failure of people to recognise weight gain. If we, or our health services, cannot accurately index body size, then the appropriate compensatory behaviours which might reduce weight will not be undertaken (Robinson, Parretti, & Aveyard, 2014).

Furthermore, inaccurate perception of body size is a key feature of anorexia and bulimia nervosa for both men and women (e.g., Dakanalis et al., 2015; Fairburn, Cooper, Doll, & Welch, 1999; Lavender, Brown, & Murray, 2017; Mitchison & Mond, 2015; Rosen, 1997). It has even been suggested that anorexia nervosa be renamed as a body image disorder (Phillipou, Castle, & Rossell, 2018). In the UK alone, the different forms of eating disorders affect at least 600,000 people and cost the UK economy £15 billion each year in treatment, reduced productivity, and reduced earning (BEAT, 2015). Body size overestimation is one of the most persistent of all the symptoms in anorexia and bulimia nervosa, predicting onset of weight-loss behaviours, and its severity predicts treatment outcomes and relapse rates (Castro, Gila, Puig, Rodriguez, & Toro, 2004; Freeman, Thomas, Solyom, & Koopman, 1985; Junne et al., 2019; Liechty, 2010). Thus, the ability to provide an accurate index of a patient’s body size perception and provide feedback on the accuracy of this judgement is a key aspect of treatment (Gledhill et al., 2017; Irvine et al., 2020).

**Fig. 1:**
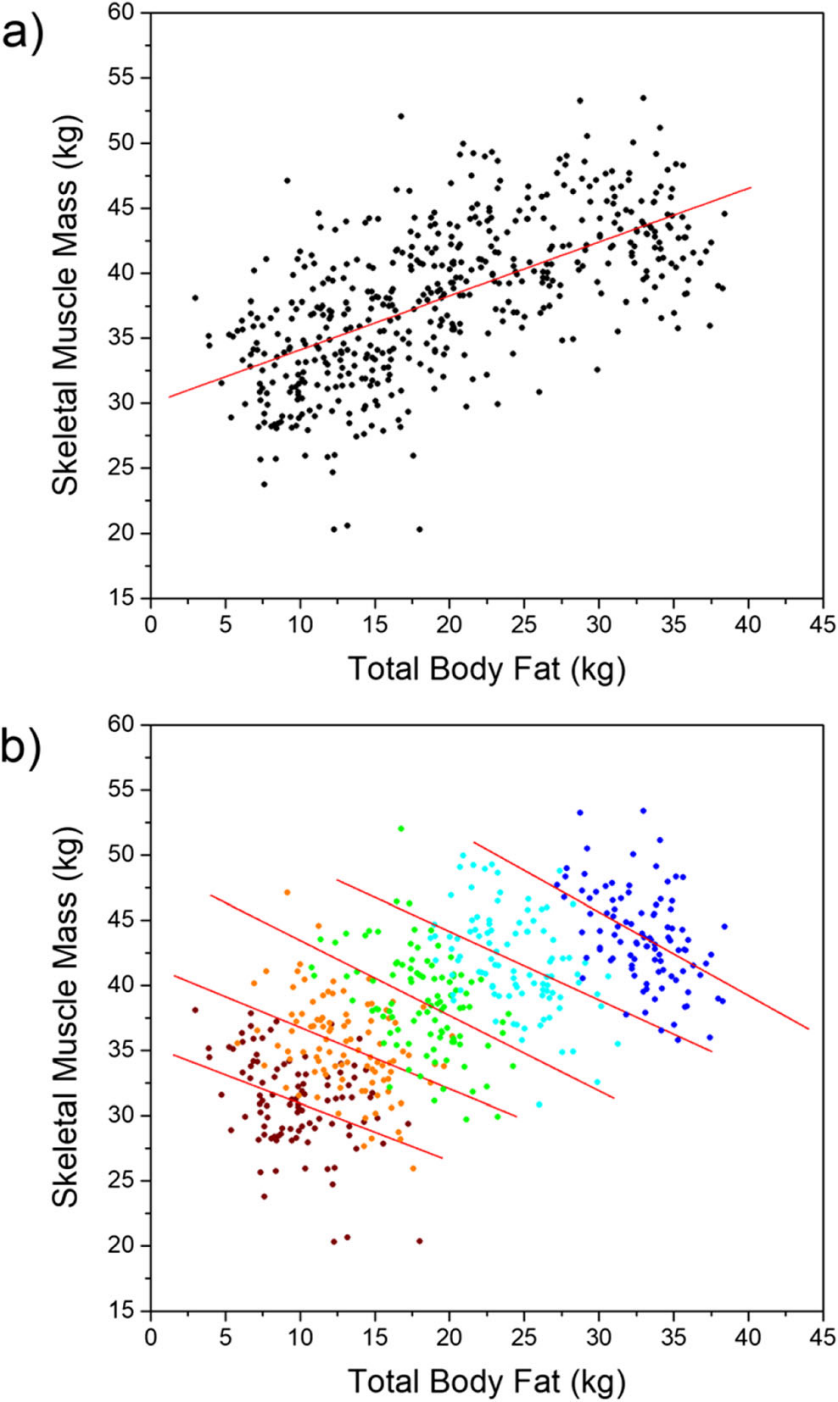
The top row (**a**) shows a simulation of the relationship between muscle mass and fat mass in 500 men. The red line is the ordinary least squares (OLS) regression of muscle mass on fat mass across the whole sample. The bottom row (**b**) shows a plot of the same data partitioned into five equally sized subgroups of 100 individuals, based on the BMI ranges 15–19 (wine), 19–23 (orange), 23–27 (green), 27–31 (cyan), and 31–35 (blue). The red lines are the OLS regression of muscle mass on fat calculated separately for each subgroup. To create this illustration, the covariance values for the relationships between BMI, fat mass, and muscle mass were taken from Groves et al. (2019)

However, existing assessment tools fail to accurately capture how bodies vary in size and shape. They make the false assumption that body mass index (BMI) is an accurate index of body fat, and on this basis attempt to simulate BMI change in a sequence of bodies (Gardner & Brown, 2010). The problem here is that body shape change is predicted by variation along two features of body composition (adiposity and muscularity) and not one (Sturman, Stephen, Mond, Stevenson, & Brooks, 2017). Indeed, the relationship between body composition and BMI represents a clear example of Simpson’s paradox, as shown in Fig. 1. This illustrates how plots of muscle mass as a function of body fat are positively correlated across any reasonably large sample of men or women. However, if the data sets are subdivided into narrow ranges of BMI, then the direction of the relationship between muscle and fat mass inverts and becomes negative. It is for exactly this reason that individuals can have the same BMI but different body composition (i.e., higher muscle mass with lower body fat, or vice versa), and therefore different body shapes (Mullie, Vansant, Hulens, Clarys, & Degrave, 2008; Yajnik & Yudkin, 2004). As a result, BMI is an inaccurate measure of body composition and misallocates people into the wrong risk categories for health risks and weight-related disease (e.g., Okorodudu et al., 2010). Additionally, the mismatch of size and shape between BMI and actual body composition introduces a significant error in the choice of which body in a sequence of bodies varying in BMI corresponds to a participant’s own body. This error may be as high as 5–7 BMI units (Groves et al., 2019), again significantly shifting the chosen body across the World Health Organization BMI categories for health risk (WHO, 2018).

To solve this problem and accurately represent the variation that exists in body size and shape, it is necessary to combine body composition measurements with 3D body shape scanning techniques in a large sample of volunteers. Such a data set can then be used to determine the statistical mapping between 3D body shape change as a function of muscle mass and adiposity independently, and these statistical models could be used in turn to create appropriately calibrated 3D computer-generated models of bodies.

Here, we report (i) the collection of a new data set combining 3D body shape scans together with bioelectrical-impedance measures of body composition; (ii) a novel analysis of these two data sets which allows a calibrated mapping between 3D body shape, muscle mass, and fat mass to generate computer-generated imagery (CGI) stimuli; (iii) the proposal of a new 2D method of adjustment task which allows participants to select a body size/shape they believe themselves to have, or would like to have, expressed as body composition (i.e., a 2D outcome variable comprising both muscle and fat mass); (iv) the presentation of a new analysis pipeline, illustrated with toy data sets, in which multivariate regression is used to map the measured body composition of the observer onto the body composition derived from our method of adjustment task.

## Methods

### 3D body shape database collection

#### Participants

Ethical permission was granted by the School of Psychology Research Ethics Committee (SOPREC) at the University of Lincoln (approval code PSY1718350). A total of 560 adults aged 18–74 years were recruited from staff and students at the University of Lincoln and the general population in and around Lincoln. We have only included data from Caucasian adults aged 18–45 in this particular analysis, as the pattern of fat deposition varies in different racial and age groups (Gallagher et al. 1996; Misra & Khurana, 2011; Wells, Cole, Brunner, & Treleaven, 2008). The final sample (*n* = 397) comprised 176 men (*M*_age_ = 28.84, *SD* = 7.99) and 221 women (*M*_age_ = 29.14, *SD* = 8.18). No screening for eating disorders was carried out, so it is possible that some participants had an eating disorder but none of them identified themselves as such. Table 1 summarises the participants’ anthropometric and body composition measurements, and Table 2 summarises the BMI category distribution of the sample, separately for men and women.

**Table 1:** Participant anthropometric and body composition measurements for men and women

	Women (*n* = 221)				Men (*n* = 176)			
	Min.	Max.	Mean	SD	Min.	Max.	Mean	SD
Height (cm)	141.50	181.00	164.63	6.29	165.00	198.50	179.41	6.80
Fat (kg)	4.20	52.90	17.65	7.92	2.50	46.00	14.53	7.39
Skeletal muscle (kg)	18.90	35.10	26.34	2.79	28.40	61.60	39.55	5.61
BMI	16.69	38.05	23.82	4.19	16.19	38.74	25.35	3.79

**Table 2:** The number and proportion of men and women in each BMI category

	Women (*n* = 221)		Men (*n* = 176)	
BMI category	Frequency	Percent	Frequency	Percent
Underweight	8	3.60	5	2.80
Normal weight	151	68.30	85	48.30
Overweight	41	18.60	66	37.50
Obese	21	9.50	20	11.40

*Note.* The BMI categories reported here are according to the World Health Organization definitions: underweight < 18.50 kg/m^2^, normal weight 18.50–24.99 kg/m^2^, overweight 25.00–29.99 kg/m^2^, and obese > 30.00 kg/m^2^

#### Equipment

##### 3dMD scanner

High-resolution, colour, 3D body scans of each participant were obtained using a 3dMD anthropometric surface imaging system. The 360° full-body scanner incorporates nine modular camera units, which are distributed around a circle approximately 4 m in diameter, with equal spacing between modules. The participant to be scanned stands in the middle of this circle. Each unit contains two monochromatic cameras and one speckle projector for capturing body geometry, and one colour camera capturing body texture. The speckle cameras automatically projected a standard light pattern onto the body when the mono cameras were capturing an image, while light-emitting diode panels were turned on when the colour camera was capturing an image. The scanner was set up to capture seven frames per second, with a total of 20 seconds required for each 3D body scan. The output from the 3dMD system included a 3D full-body polygon surface mesh with X, Y, and Z coordinates, as well as a mapped surface texture. Geometric accuracy for this system is approximately 0.5 mm or below (3dMD, 2019).

##### Tanita body composition analyser

Body composition measurements were obtained using a Tanita MC-780MA multi-frequency segmental body composition analyser. This device uses eight-electrode bioelectrical impedance analysis (BIA) to send a weak, undetectable electrical current through the body to estimate a person’s body composition using a high-frequency current (50 kHz, 90 μa). The scale outputs total body measurements of body fat, skeletal muscle, visceral fat rating, water content, bone mass, BMI, and basal metabolic rate. Separate body fat and muscle (mass and percentage) estimations for individual segments of the body, including the central trunk, right arm, right leg, left arm, and left leg, are also outputted. The outputs of the device are calibrated for the sex, age, and height of the individual being measured, with this information being entered by the operator. The results obtained with the Tanita bio-electrical impedance analysis have been shown to be within **±**5% of underwater weighing and dual-energy X-ray absorptiometry (DEXA), the ‘gold’ standards of body composition analysis) (Völgyi et al. 2008; Sillanpää et al., 2014).

##### Procedure

**Fig. 2:**
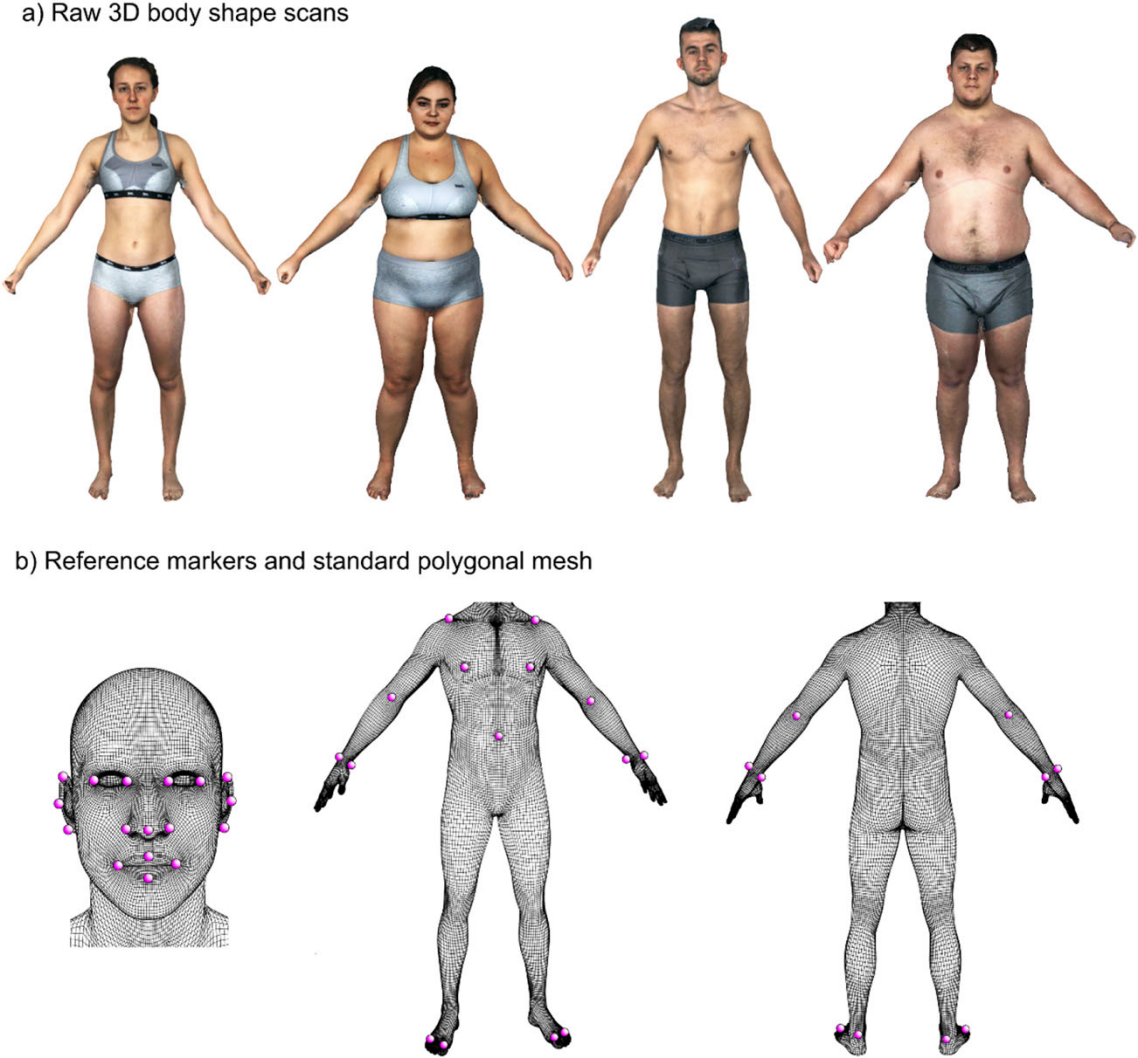
The top row (**a**) presents two female and two male 3D body scans prior to Wrap 3 processing. The bottom row (**b**) shows the template base mesh with 36 preselected landmarks

Participants were first scanned using the 3dMD body scanner. During the 20 s scan, participants were asked to stand in the centre of the space around which the cameras were distributed, with their feet shoulder-width apart. To capture a range of arm positions, participants were asked to slowly raise their arms to shoulder level with their hands in a fist. Participants were provided with tight-fitting, grey underwear in a range of sizes to ensure that body shape was not disguised by clothing. Men were asked to wear boxer-style shorts while women wore a sports bra and shorts (see Fig. 2). Next, standing height was measured (to the nearest centimetre) using a stadiometer after participants were instructed to stand up straight and face forward. Lastly, body composition measurements were taken using the Tanita body composition analyser. This process lasted approximately 20 minutes.

##### Scan processing

A suitable frame from each 20-second scan was selected using 3dMD software prior to processing of the scans. This frame was chosen to depict the individual standing with their arms away from the body in an ‘A-pose’. The 3D scans were then processed using Wrap3 software (version 3.3.17, Russian3DScanner, 2018) in order to repair any missing segments and remove any non-manifold topology or irrelevant components from each scan. A template base mesh was wrapped around the individual scans by matching 36 preselected points (manually located) on corresponding landmarks of both the 3D scan and template model (see Fig. 2). This resulted in all scans having a standardised topology, allowing for statistical comparisons to be made whilst maintaining individual variation in body size and shape. Polygon selection was used to exclude the hands of each scan from wrapping, as this feature was not relevant to the data analysis. Each processed scan consisted of 79,522 vertices.

##### Body composition reliability and validity

Bioelectrical impedance analysis (BIA) is a relatively inexpensive, easy-to-use, and quick method for estimating body composition which is less prone to technical error than other methods, making it a suitable tool for large-scale studies (Lee & Gallagher, 2008). Multiple studies have found BIA to be a valid tool for estimating body fat in adults. This technique shows good agreement compared to dual-energy X-ray absorptiometry (e.g. Ling et al., 2011; Sun et al., 2005; Wattanapenpaiboon, Lukito, Strauss, Hsu-Hage, Wahlqvist, & Stroud, 1998) and skinfold calliper measurements (Kitano, Kitano, Inomoto, & Futatsuka, 2001). Furthermore, BIA shows good test–rest reliability (Aandstad, Holtberget, Hageberg, Holme, & Anderssen, 2014; Jackson, Pollock, Graves, & Mahar, 1988). Here, we report reliability and validity data for the body composition measurements taken in this sample.

##### Validation of body fat measurements from BIA

To validate body fat measurements taken from the BIA in this sample, skinfold measurements were taken by a Level 2 International Society for the Advancement of Kinanthropometry (ISAK) practitioner for a subset of participants (26 men and 22 women) using standard ISAK techniques (Stewart, Marfell-Jones, Olds, & De Ridder, 2011). Skinfold measurements were taken from eight skinfold sites—tricep, bicep, subscapular, iliac crest, supraspinale, abdominal, medial calf, and front thigh—using skinfold callipers (Harpenden, HaB, UK). The mean of two measurements was used unless values differed by ≥ 5%, whereupon a further skinfold measure was taken, and the median value was used. The following four-site skinfold equations (Jackson & Pollock, 1985) were then used to estimate percentage body fat, based on the abdominal, tricep, front thigh, and iliac crest skinfolds:

$$ \mathrm{Men}:\left(0.29288\times \mathrm{sum}\ \mathrm{of}\ \mathrm{skinfolds}\right)-\left(0.0005\times \mathrm{square}\ \mathrm{of}\ \mathrm{the}\ \mathrm{sum}\ \mathrm{of}\ \mathrm{skinfolds}\right)+\left(0.15845\times \mathrm{age}\right)-5.76377 $$

$$ \mathrm{Women}:\left(0.29669\times \mathrm{sum}\ \mathrm{of}\ \mathrm{skinfolds}\right)-\left(0.00043\times \mathrm{square}\ \mathrm{of}\ \mathrm{the}\ \mathrm{sum}\ \mathrm{of}\ \mathrm{skinfolds}\right)+\left(0.02963\times \mathrm{age}\right)+1.4072 $$

Estimates of total fat mass were also calculated based on participants’ total body weight and their estimated percentage body fat from the Jackson and Pollock (1985) equations.

Pearson’s correlations were used to explore the relationship between fat estimates taken from the calliper method (body fat percentage and fat mass in kilograms) and BIA (body fat percentage and body fat mass in kilograms), separately for men and women. The results shown in Table 3 indicate that the body fat values derived from the callipers and BIA were significantly, positively correlated for both samples of men and women.

**Table 3:** Correlations between BIA and calliper estimates of fat mass and percentage for men and women

	Women (*n* = 22)		Men (*n* = 26)	
	Calliper body fat %	Calliper fat mass	Calliper fat %	Calliper fat mass
BIA fat %	0.76***	0.77***	0.71***	0.76***
BIA fat mass	0.80***	0.89***	0.60***	0.74***

***p < .001

**Fig. 3:**
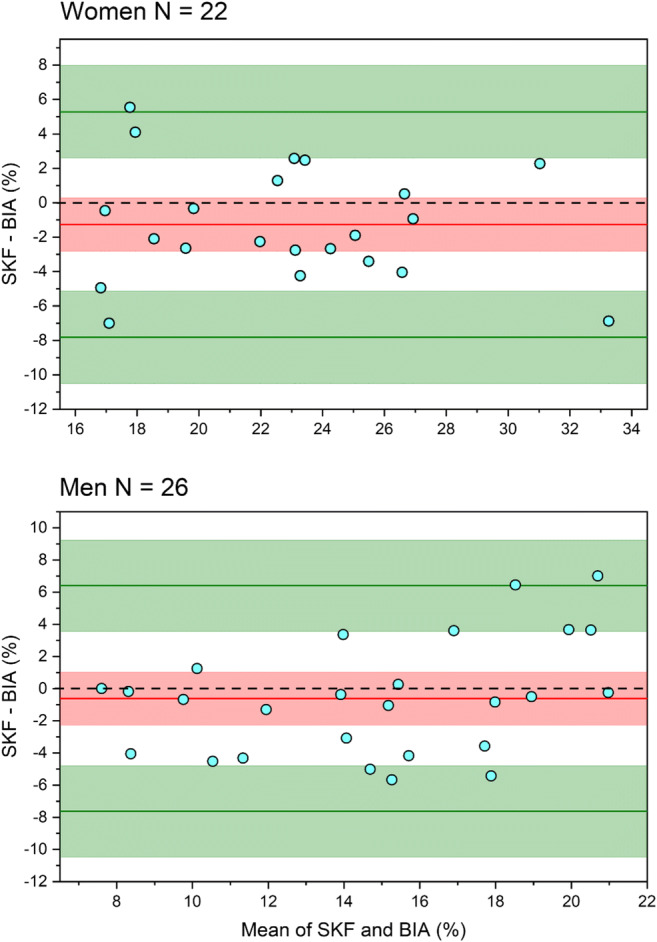
Bland-Altman plots of the differences between BIA and calliper estimates of percentage body fat. The mean difference is −0.61 and −1.27 for men and women, respectively. The limits of agreement are denoted by the upper and lower solid lines representing the 95% CIs

The body fat percentage estimates from the calliper (*M*_women_ = 22.15, *SD* = 4.59; *M*_male_ = 14.55, *SD* = 5.04) and BIA (*M*_women_ = 23.42, SD = 5.03; *M*_male_ = 15.16, *SD* = 3.81) were not significantly different, for both men *t*(25) = −0.87, *p* = .395 and women *t*(21) = −1.78, *p* = .090. This good agreement is illustrated by the Altman-Bland plots between BIA and calliper estimates in Fig. 3, and is consistent with previous studies (see e.g., Kitano et al., 2001; Wattanapenpaiboon et al., 1998).

##### BIA intra-individual reliability

To assess the reliability of the BIA, repeat measurements were taken from a subset of participants (9 women; *M*_BMI_ = 21.88, *SD* = 2.09) during the same sessions in which they took part. A Pearson’s correlation was calculated in order to investigate the relationships between body composition variables (fat mass, fat percentage, muscle mass, and fat-free mass) at the two time points. All body composition values at T1 and T2 were significantly, positively correlated (*r* > .99, *p* < .001). Comparison between the measurements at both time points demonstrated excellent agreement, with the intraclass correlation coefficient (ICC) for each variable being greater than .99 (*p* < .001).

### Mapping 3D body shape onto body composition

#### Body shape

Using customised MATLAB software, we excluded the 3D coordinates associated with points referring to the head, neck, hands, and feet in the processed scans. The remaining 26,665 coordinates described the legs, arms, and torso. The average 3D shape for the set was then calculated, and all individual shapes were subsequently fitted to this average using Procrustes analysis in order to minimise idiosyncratic differences in body position. It is important to note that only translation and orthogonal rotation were utilised in order to preserve those aspects of shape change related to scaling (i.e., size).

**Fig. 4:**
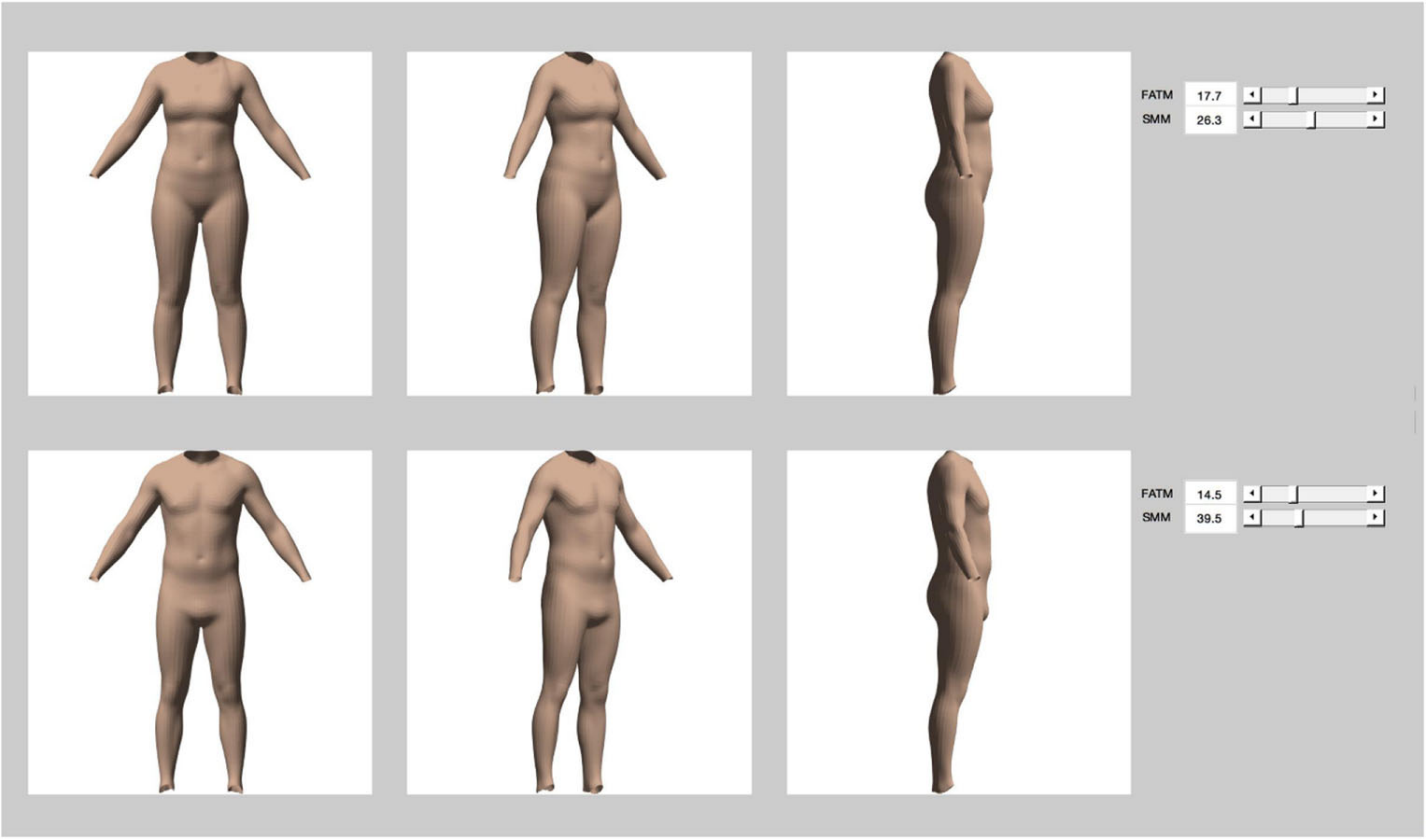
The visualisation tool allows body shape to be predicted for a given pair of FATM and SMM values. The top row shows the average female body shape and the bottom row shows the average male body

Next, each individual shape was converted to a vector of 79,995 numbers (26,665 points × 3 coordinates), with these vectors entered into a principal component analysis (PCA). The resulting subspace comprised *c* − 1 dimensions, where *c* is the number of identities. For each dimension in the subspace separately, we carried out a linear regression. All identities’ measures of fat mass (FATM) and skeletal muscle mass (SMM) taken from BIA were used to predict their locations along that specific dimension, with the values of the two coefficients and the constant subsequently allowing us to model shape change. It was not important to consider whether these regressions were statistically significant, since each simply described the relationship between the two body measures and shape for a given subspace dimension—if no relationship existed, then the coefficients would be small, and their effect on shape change in the model would reflect this. Using the results of these regressions, we were therefore able to predict locations along all subspace dimensions for any given pair of FATM and SMM values. For the specific location identified in multidimensional space, the 3D shape could then be reconstructed and visualised (see Fig. 4).

**Fig. 5:**
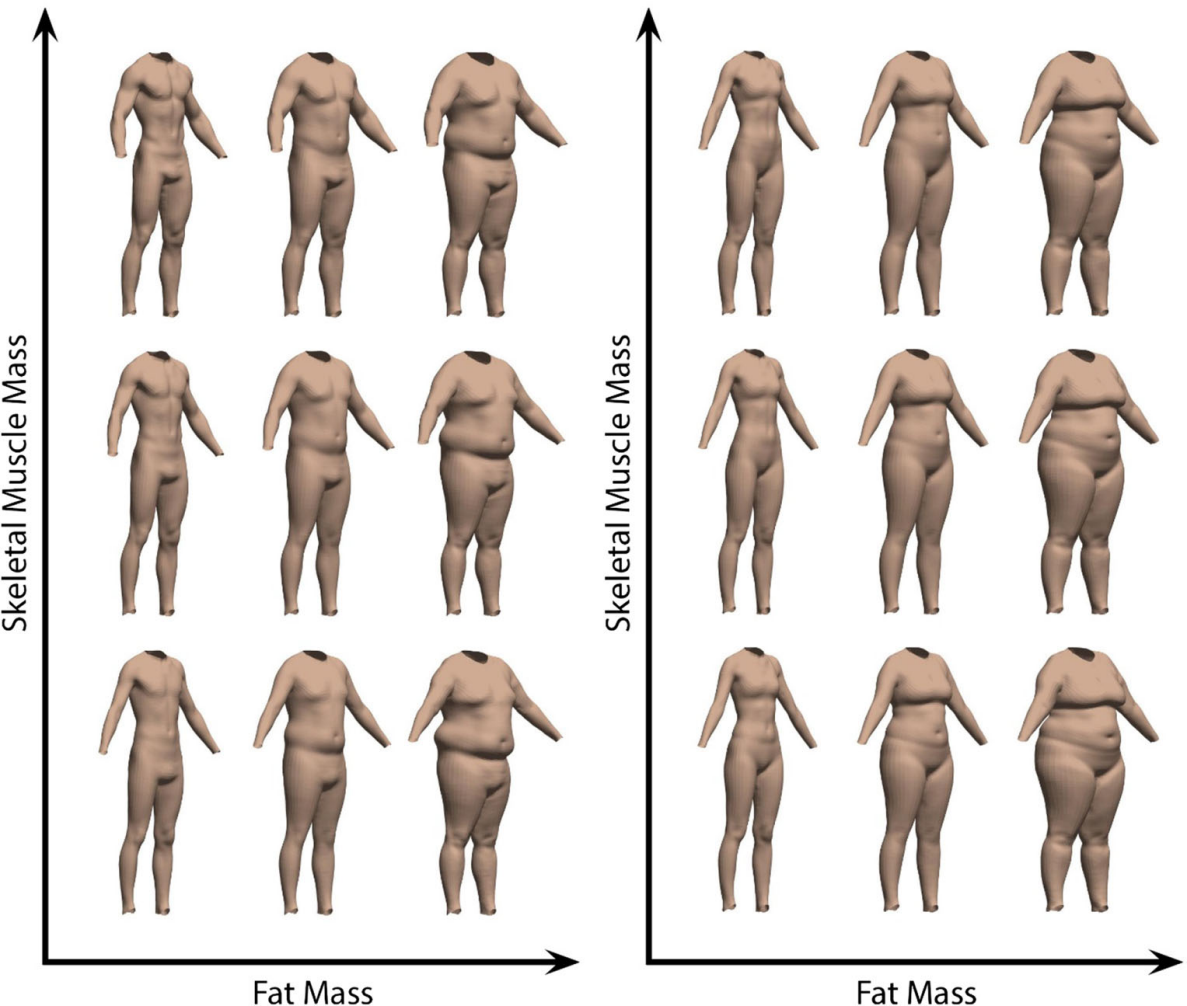
Visualisations of male (left) and female (right) predicted body shape at the highest, middle, and lowest values of FATM and SMM for our sample

Given that our model of shape change was derived from a specific database of 3D scans (representing typical population values of both FATM and SMM), we chose to only consider and discuss our predictive model within the limits of the actual values of our sample. In other words, we did not explore how body shape might vary outside of the lowest and highest values that were measured in our identities (see Fig. 5).

#### Comparing our model to predictions based on BMI

For bodies within our sample, we investigated how well the model was able to predict body shape in comparison with BMI. To do this, we utilised a ‘leave-one-out’ strategy in order to determine how novel test shapes could be predicted from a sample of training shapes. We cycled through each identity, removing their 3D scan from the sample and using the remaining identities’ scans in the ‘PCA + regressions’ model of shape change described above. In addition to our FATM/SMM model, we separately modelled shape change using the BMI values of our identities. (As above, training identities’ measures of BMI were used to predict their locations along each PCA dimension, with the values of the coefficient and the constant allowing us to model shape change.)

The excluded identity’s scan was then compared with the predicted 3D shape for that identity based on their measures of FATM and SMM, and separately, the predicted 3D shape based on their measure of BMI. In order to quantify error when comparing these predicted shapes with the original scans, we calculated the ‘straight line’ distance in 3D space between every original point and its predicted location, subsequently averaging these distances across all points. Here, we considered only the 12,697 points representing the torso, which allowed us to remove prediction errors inherent in the arms and legs as a result of their positioning. (While standard instructions were given to participants during scanning, no constraints were placed on the locations of the feet and hands in the resulting scans.)

For every identity, we therefore calculated this measure of error when predicting 3D shape (excluded from the sample used in deriving the models) from FATM and SMM, and separately, from BMI. For our male sample, a paired-samples *t* test comparing these two measures of error confirmed that our FATM/SMM model (*M* = 1.71, *SD* = 0.49) performed better than the BMI model (*M* = 1.83, *SD* = 0.56), *t*(175) = 5.83, *p* < .001, Cohen’s *d* = 0.44. This result was also found for our female sample (FATM/SMM model − *M* = 1.59, *SD* = 0.51; BMI model − *M* = 1.71, *SD* = 0.57), *t*(220) = 5.18, *p* < .001, Cohen’s *d* = 0.35. In other words, for both men and women, we were better able to predict 3D shape using a model incorporating FATM and SMM in comparison with one based on BMI.

**Fig. 6:**
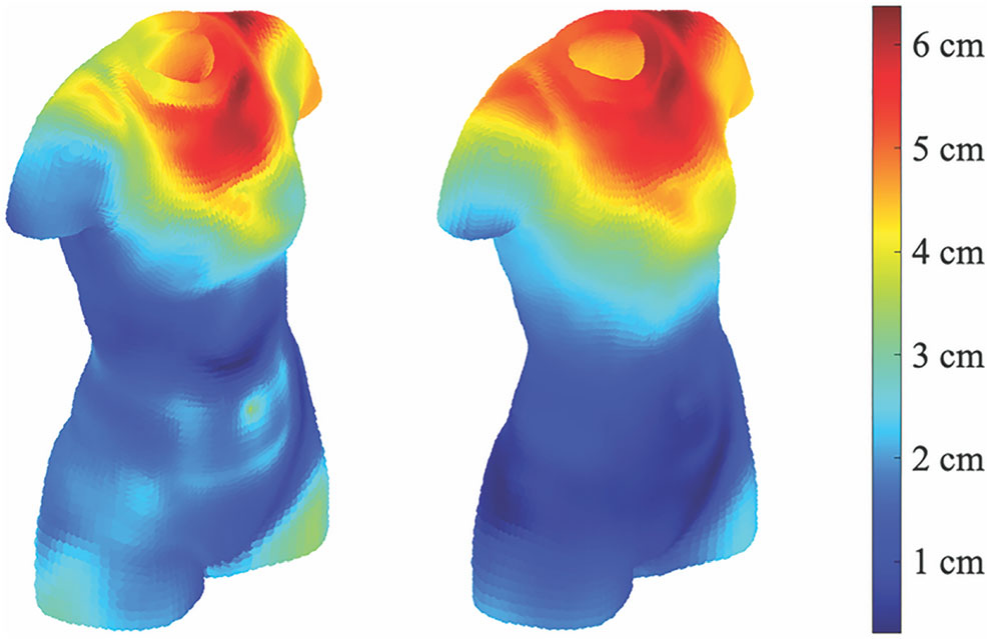
Displaying prediction errors for an example female identity’s 3D shape. Errors for the FATM/SMM (left) and BMI (right) models are shown, with warmer-coloured points representing larger errors in prediction for this shape

**Fig. 7:**
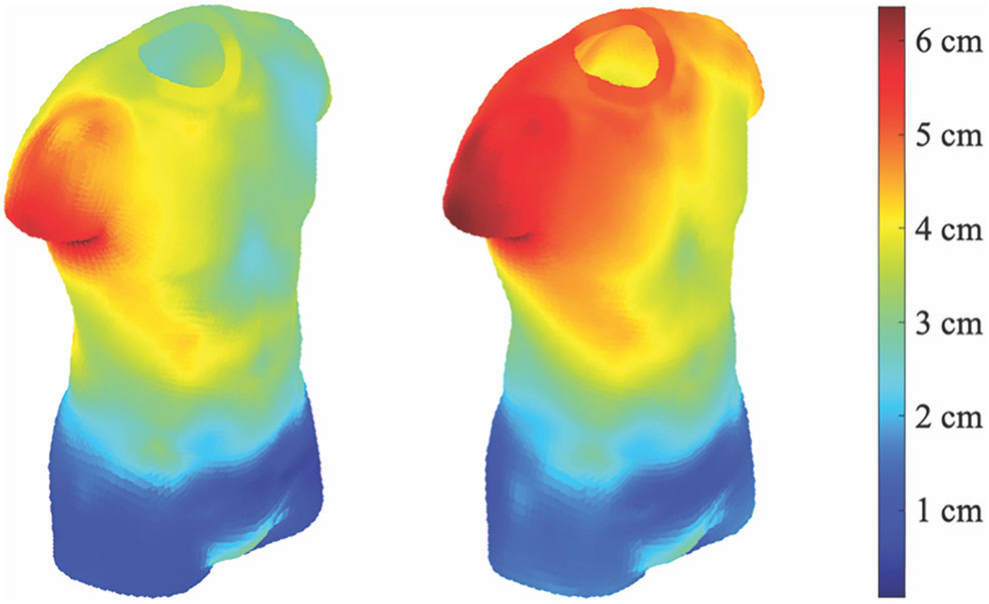
Displaying prediction errors for an example male identity’s 3D shape. Errors for the FATM/SMM (left) and BMI (right) models are shown, with warmer-coloured points representing larger errors in prediction for this shape

Figures 6 and 7 illustrate this result by displaying the errors in shape prediction for two specific identities (a woman and a man, respectively), comparing the predicted 3D shapes of the two models beside each other. In order to generate these displays, we found the maximum error for all points across both models for the identity featured, and then converted prediction errors for each point to be a proportion of this maximum. (Across all identities: average female maximum error, *M* = 4.36 cm, *SD* = 2.76 cm; average male maximum error, *M* = 4.29 cm, *SD* = 1.11 cm.) As such, increasingly warm-coloured points in the figures represent larger errors on the same scale. For the examples illustrated in Figs. 6 and 7, the larger errors for the BMI model (displayed on the right-hand side in both figures) appear to be concentrated, for the most part, in the upper torso. As can be seen, the errors for the BMI model are greater for the male example, reflecting the greater variation in fat and muscle in men which the unidimensional BMI model cannot accurately capture.

#### Predicting individual changes

**Fig. 8.**
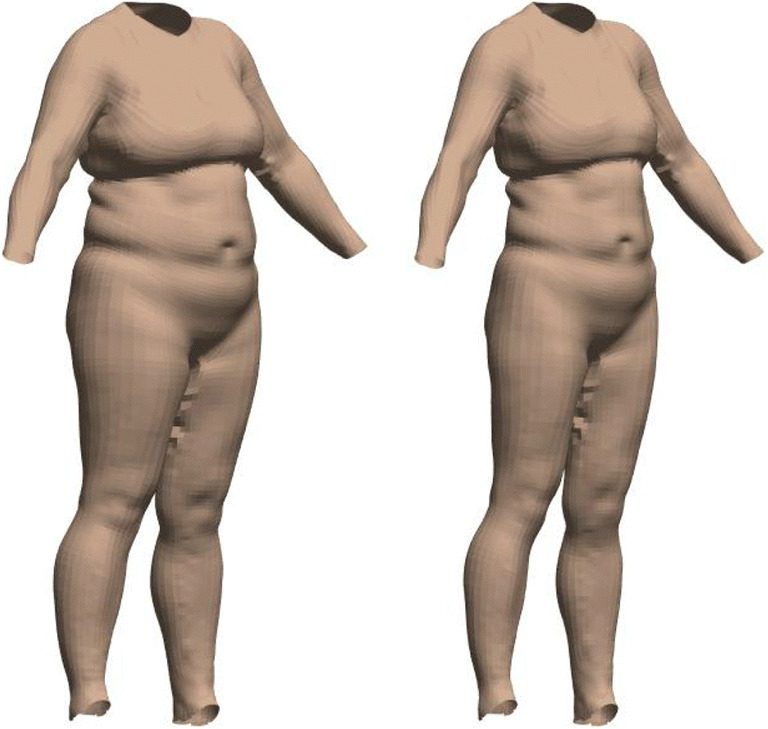
Predicting shape change for a specific woman. The original 3D scan (left; FATM = 26.6 kg, SMM = 32.7 kg), and how the woman’s shape is predicted to change if she halved her FATM (right)

**Fig. 9:**
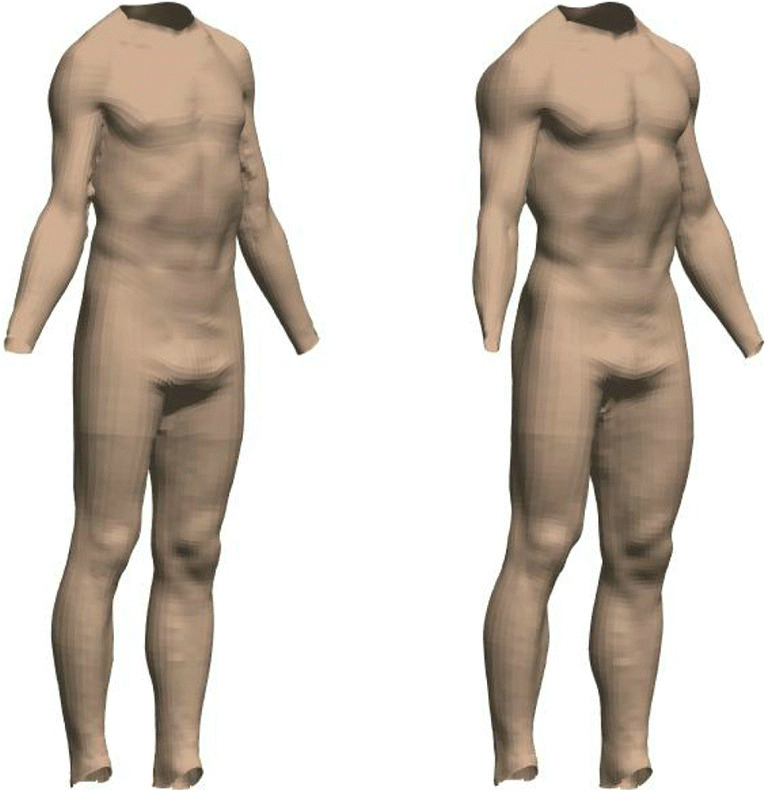
Predicting shape change for a specific man. The original 3D scan (left; FATM = 7.0 kg, SMM = 29.9 kg), and how the man’s shape is predicted to change if he doubled his SMM (right)

Above, we described our model of shape change based on FATM and SMM, and how this was able to predict body shape for a given pair of values. However, this modelling process can also be used to predict how a given individual’s body shape would change with an increase or decrease in fat and muscle values. We simply generate the model for FATM/SMM described above (PCA + regressions) and then apply the predicted changes to shape that are associated with a change in these two measures. Rather than visualising these shifts along the various principal components in terms of the average body shape (above), our starting point in the multidimensional space is the individual’s shape itself. As such, predicted shape changes are applied to a specific person, enabling data-driven predictions regarding how an individual might vary (see Figs. 8 and 9).

#### Behavioural task

In order to obtain judgements of body size/shape from participants, we will use the method of adjustment. The task will be designed so that the fat and muscle mass of a CGI model stimulus presented on a PC monitor can be manipulated smoothly, in real time. Using two sets of arrow buttons on the screen, participants will be able to systematically change the fat and muscle mass of the stimulus. On each trial in the task, the CGI model will be assigned an arbitrary fat and muscle mass combination as a start point. The job of the participant will be to modify the CGI model so as to best capture the body size/shape that they believe themselves to have, if making self-estimates of body size, or would like to have, if making estimates of ideal body size/shape. Once the participant is satisfied with their choice of body composition on each trial, they will press a response button which will allow the muscle and fat mass combination for that trial to be recorded, and a new trial initiated.

**Fig. 10:**
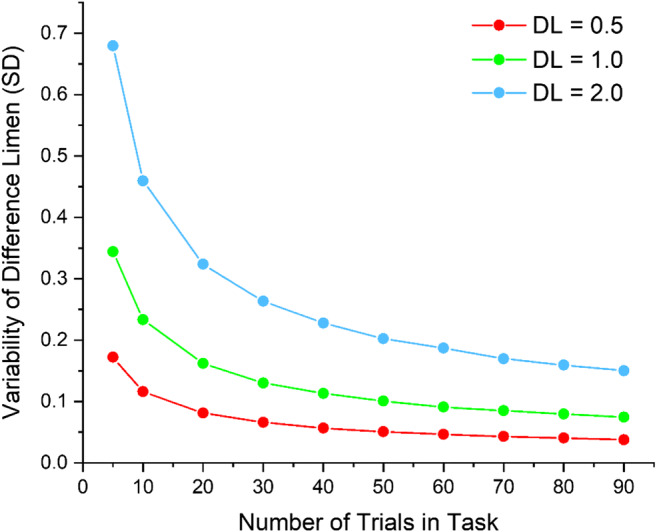
Plots of the variability in DL estimates as a function of the number of trials in the method of adjustment task

According to classical psychophysics (Gescheider, 1997), the mean of the muscle and fat mass values, respectively, will represent an estimate of the point of subjective equality (PSE) for the body composition that the participant believes they have, or would like to have (depending on task instruction). Moreover, the standard deviations of these means represent the difference limen (DL), a measure of task sensitivity or precision. Figure 10 shows a Monte Carlo simulation to estimate the variability in DL estimates as a function of the number of trials in the method of adjustment task. The simulation was run for target DL values of 0.5, 1.0, and 2.0. These were to be estimated from tasks comprising 5, 10, 20, 30, 40, 50, 60, 70, 80, and 90 trials. Each data point in Fig. 10 is derived from 10,000 resamples. It shows an elbow region around 20–60 trials per participant, suggesting that around this number should be sufficient to obtain stable estimates of DL.

#### Behavioural data analysis

The Pearson correlations between measured fat mass and muscle mass in the men and women who agreed to be 3D body scanned were *r* = 0.45, *p* < .001, and *r* = 0.38, *p* < .001, respectively. This means that the fat and muscle mass values obtained from the estimates of body composition, in the method of adjustment task, are also highly likely to be correlated. If they were not correlated, then we could model the fat and muscle components of participants’ responses using separate multiple regression models. Here, we assume this is unlikely to be the case. Therefore, to map the relationships between the body composition that participants actually have versus the body composition they think they have (or would like to have), we will need to use multivariate regression.

The standard multivariate linear model can be written as *Y = XB + E*. *Y* is an *n* × *r* matrix of *r* response variables measured on *n* subjects; *X* is an *n* × *p* matrix of explanatory variables; *B* is a *p* × *r* matrix of regression coefficients; and *E* is an *n* × *r* ‘error’ matrix whose rows are independent and identically normally distributed with mean 0 and covariance matrix *Σ*. Below is a simple example with two responses and one explanatory variable (in addition to an intercept term) measured on three subjects.

$$ \left(\begin{array}{cc}Y{1}_1& Y{2}_1\\ {}Y{1}_2& Y{2}_2\\ {}Y{1}_3& Y{2}_3\end{array}\right)=\left(\begin{array}{cc}1& {X}_1\\ {}1& {X}_2\\ {}1& {X}_3\end{array}\right)\left(\begin{array}{cc}{\beta}_{01}& {\beta}_{02}\\ {}{\beta}_{11}& {\beta}_{12}\end{array}\right)+\left(\begin{array}{cc}\varepsilon {1}_1& \varepsilon {2}_1\\ {}\varepsilon {1}_2& \varepsilon {2}_2\\ {}\varepsilon {1}_3& \varepsilon {2}_3\end{array}\right) $$

Here we used PROC MIXED in SAS (v9.4) to implement two multivariate regressions of toy data sets intended to represent the kinds of responses we might expect from body composition estimates using the 2D method of adjustment task (see also Wright, 1998). In both cases, we have as explanatory variables: (i) participants’ measured fat mass, (ii) participants’ measured skeletal muscle mass, and (iii) a psychometric covariate related to participants’ attitudes and behaviours about muscularity. To simulate the two outcome variables from the method of adjustment task in men, i.e., estimated muscle mass and estimated fat mass, we assume a covariance between the two measured participant muscle and fat masses of 0.45, and covariances between the psychometric covariate and measured fat and muscle masses of 0 and 0, respectively.

**Fig. 11:**
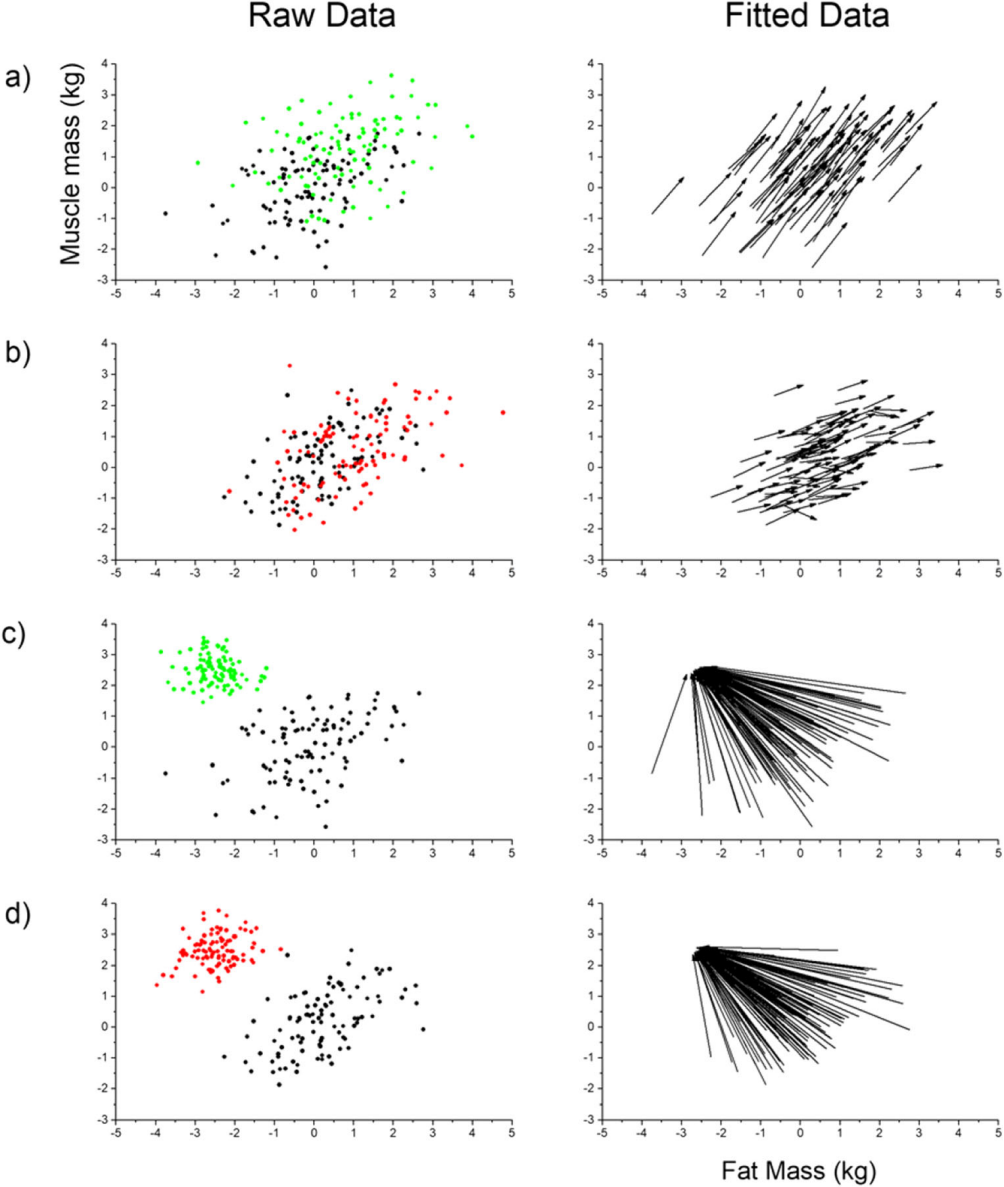
Both the left and right columns represent plots of muscle mass (*y*-axis) as a function of fat mass (*x*-axis). All values are in *z*-scores. Rows (**a**) and (**b**) correspond to the first simulation of self-estimates of body size/shape. Rows (**c**) and (**d**) correspond to the second simulation of ideal estimates of body size/shape. The left column represents the raw data. In each row, black dots represent the measured body composition of participants. The coloured dots represent the responses from the method of adjustment task; green dots correspond to individuals with psychometric scores in the highest 30%, and red dots individuals with psychometric scores in the lowest 30%. The right column is a set of vector plots which join the measured body composition of an individual (arrow start) to the body composition predicted from the multivariate models (arrow end)

The first scenario is one in which male participants were asked to estimate their own body composition. In this simulation, we assumed that they overestimated both their fat and muscle masses by, on average, 1 unit (see Table 4 for summary of parameter values). We also allowed an additional, statistically independent contribution to the muscle mass estimate from the psychometric task: higher scores on this task were associated with higher estimates of muscle mass. In the second scenario, male participants were asked to estimate their ideal body composition. For this simulation, we assumed that participants’ psychometric performance was unrelated to their responses, and that all participants tended to converge on a common ideal with low body fat and high muscle mass. The individual simulation parameters, their estimates derived from multivariate regression, and the overall multivariate analysis of variance (MANOVA) statistics are shown in Table 4. In addition, these results are plotted in Fig. 11.

**Table 4:** Comparison between simulation and modelled parameters

	Self-estimate of body size/shape				
Outcome variable	Explanatory variable	Simulation parameter	Modelled parameter	Wilks’ lambda	*p*
Estimated fat	Intercept Ppt Fat Ppt Muscle Psych	1 1 0 0	1.0065 1.0073 −0.023 0.0023		<.001 <.001 .19 .88
Estimated muscle	Intercept Ppt Fat Ppt Muscle Psych	1 0 1 0.5	0.96 0.016 0.96 0.51		<.001 .36 <.001 <.001
MANOVA	Ppt Fat Ppt Muscle Psych			0.23 0.24 0.47	<.001 <.001 <.001
	Ideal estimate of body size/shape				
Outcome variable	Explanatory variable	Simulation parameter	Modelled parameter	Wilks’ lambda	*p*
Estimated fat	Intercept Ppt Fat Ppt Muscle Psych	−2.5 0.1 0 0	−2.49 0.11 −0.023 0.0023		<.001 <.001 .19 .88
Estimated muscle	Intercept Ppt Fat Ppt Muscle Psych	2.5 0 0.1 0	2.46 0.016 0.056 0.015		<.001 .36 .001 .34
MANOVA	Ppt Fat Ppt Muscle Psych			0.96 0.99 0.99	<.001 .002 .63

*Note.* Ppt Fat = measured fat mass of participant; Ppt Muscle = measured muscle mass of participant; Psych = psychometric covariate reflecting attitudes and behaviours around muscularity

## Discussion

A fundamental problem with previous sets of images used to test judgements of body size and shape is biometric validity. That is to say, the size and shape of the bodies used are a poor fit to the actual physical characteristics of the bodies they are intended to represent. Part of this problem is the reliance on trying to represent body shape as predicted by BMI. In reality, as illustrated in Fig. 1, bodies with the same BMI may have very different body composition, and this is reflected in different body shapes and sizes. Therefore, any body size/shape estimation task that relies on participants matching their beliefs (or desires) against stimuli calibrated only for BMI is bound to be error-prone (see Groves et al., 2019). Attempts have been made to address this issue by constructing body scales which systematically vary combinations of muscle mass and adiposity (e.g., Cafri & Thompson, 2004; Talbot, Smith, Cass, & Griffiths, 2019). However, these assessment tools do not provide a calibrated mapping between the size and shape of the test bodies and their composition. As a result, they cannot be used to reliably assess the perception of body shape and size (Groves et al., 2019). To directly address this problem, we are developing a new assessment tool for body size/shape estimation. In this task, we ask participants, effectively, to identify the body composition they think they have (or would like to have) using CGI stimuli correctly calibrated for both muscle and fat mass.

To create this assessment tool, we have collected measures from 176 men and 221 women who consented to have both their 3D body shape and body composition measured. This allowed the construction of a statistical model that maps 3D shape onto composition, and we demonstrate how multivariate regression can then be used to analyse what is now a 2D outcome variable. Further development work to refine the test is needed. This includes expanding the range of body shapes in the anthropometric database. Specifically, we need to sample from the edges of the body muscle/fat space, i.e., men and women who have (i) both very low muscle and fat mass, (ii) very low fat and very high muscle mass, and (iii) very high fat and low muscle mass. We also need to develop models of body shape change for people of non-European origin. The pattern of fat deposition varies in different racial groups (Misra & Khurana, 2011; Wells, Cole, Brunner, & Treleaven, 2008). For example, people of Asian and South Asian descent seem to have a higher level of fat to muscle ratio for a particular BMI, and are more likely to deposit visceral rather than subcutaneous fat on the body, resulting in different cut-offs for a healthy BMI (Shiwaku, Anuurad, Enkhmaa, Kitajima, & Yamane, 2004; WHO Expert Consultation, 2004). This difference in body composition and pattern of fat deposition underlines the need for separate databases and statistical models for different racial groups to accurately represent how body size and shape vary with changing adiposity or muscularity.

In the introduction, we briefly reviewed the literature which suggested that BMI is actually a poor predictor of body size and shape, as it did not distinguish between bodies varying in muscle content or those varying in fat content (e.g., Mullie et al., 2008; Yajnik & Yudkin, 2004). We suggested that substituting BMI for indices of muscle and fat content would provide a more accurate prediction of body shape. We further suggested that the improvement in predicting body size and shape would be greater for male bodies, as they have a wider variation in muscle and fat ratios than that for female bodies. Our analysis showed this to be the case, as the deviations from the predicted body shape were greater for BMI, and were greater for male than female bodies (visualised in Figs. 6 and 7). However, for both men and women, the FAT/SMM model performed significantly better at predicting 3D body size/shape than the BMI-only model. This emphasises the need to use body composition rather than BMI to accurately index variation in body size and shape in future research.

As discussed in the introduction, the ability to generate bodies which independently vary in either adiposity or muscularity has considerable application within health research. The biometrically accurate representation of adipose change is important for assessing body size overestimation in women with eating disorders such as anorexia and bulimia nervosa (e.g., Cornelissen, McCarty, Cornelissen, & Tovée, 2017; Gardner & Bokenkamp, 1996; Probst, Vandereycken & Van Coppenolle, 1997; Slade & Russell, 1973; Tovée, Benson, Emery, Mason, & Cohen-Tovée, 2003), where the level of body fat is believed to be a key component of their pathology (Dakanalis et al., 2015; Fairburn et al., 1999; Lavender et al., 2017; Mitchison & Mond, 2015; Rosen, 1997). This includes a more realistic simulation of body fat change in interventions for body image disturbance (Gledhill et al., 2017). It is hypothesised that the more realistic the images are in the intervention and the greater the identification of people undertaking this training, the stronger the intervention effect will be in treating their condition (Irvine et al., 2020). Additionally, with the rise of fitspiration and the importance of a toned, muscular body in the female ideal, this analysis allows the creation of stimuli with properly calibrated and independently varied fat and muscle to test the perception of this ideal (Groves et al., 2019).

We also show that the model can be applied to a specific individual’s scanned body. Previous studies have used a model based on BMI to modify an individual’s body size and shape over a limited range of ±20% in a virtual reality (VR) environment to allow women with anorexia nervosa to estimate their actual and ideal body size (Mölbert et al., 2018). Their technique’s relatively limited range of body size changes may restrict the choices that a participant could potentially make, and thus skew their results. As our new analysis is based on body composition, it enables not only a more accurate shape change but also a potentially wider range, and can be applied to CGI bodies for both conventional 2D and VR paradigms to assess body image disturbance and for interventions (such as Cornelissen, Bester, Cairns, Tovée & Cornelissen, 2015; Cornelissen et al., 2017; Irvine et al., 2020). The linear model means that the shape change can be extended over a large range and is limited only by the reliability of the predicted body shapes at the extreme ends of the spectrum. As it is a linear model, it is of course possible to extend beyond the range of body shape that we have scanned. However, extending the model beyond this range is limited by the fact that its accuracy cannot be verified. This, in turn, will be addressed by further scanning of bodies with very high or low muscle and/or fat composition to extend the body database on which the model is based.

Obviously, the next step in the development of the model is to apply the muscle and adipose dimensions to a whole body (including head, hands, and feet) with the high-resolution photographs of the body mapped onto the 3D surface (as shown in Fig. 2). This personalised approach to body perception, which could be used in either 2D or in VR, would improve the realism of the assessment of a person’s body image and the potential effectiveness of intervention paradigms (Gledhill et al., 2017; Irvine et al., 2020).

In conclusion, we have demonstrated proof of concept for a new way to obtain self-estimates of body size/shape. This method requires participants to match their beliefs/desires against CGI stimuli which have been calibrated for skeletal muscle mass and total body fat. In this way, we obtain a 2D outcome measure, body composition, the use of which avoids the confounds inherent in the alternative, BMI.

### Open practices statement

The analysis script and simulated scan data for 100 male and 100 female bodies to test the analysis are available at the Open Science Framework (https://osf.io/c6z3j/).
